# Dissecting the Relation between a Nuclear Receptor and GATA: Binding Affinity Studies of Thyroid Hormone Receptor and GATA2 on TSHβ Promoter

**DOI:** 10.1371/journal.pone.0012628

**Published:** 2010-09-07

**Authors:** Ana Carolina Migliorini Figueira, Igor Polikarpov, Dmitry Veprintsev, Guilherme Martins Santos

**Affiliations:** 1 Instituto de Física de São Carlos, Universidade de São Paulo, São Carlos, São Paulo, Brazil; 2 Biomolecular Research Laboratory, Paul Scherrer Institut, Villigen PSI, Switzerland; 3 Medical Research Council - Laboratory of Molecular Biology, Cambridge, United Kingdom; National Institute on Aging, National Institutes of Health, United States of America

## Abstract

**Background:**

Much is known about how genes regulated by nuclear receptors (NRs) are switched on in the presence of a ligand. However, the molecular mechanism for gene down-regulation by liganded NRs remains a conundrum. The interaction between two zinc-finger transcription factors, Nuclear Receptor and GATA, was described almost a decade ago as a strategy adopted by the cell to up- or down-regulate gene expression. More recently, cell-based assays have shown that the Zn-finger region of GATA2 (GATA2-Zf) has an important role in down-regulation of the thyrotropin gene (TSHβ) by liganded thyroid hormone receptor (TR).

**Methodology/Principal Findings:**

In an effort to better understand the mechanism that drives TSHβ down-regulation by a liganded TR and GATA2, we have carried out equilibrium binding assays using fluorescence anisotropy to study the interaction of recombinant TR and GATA2-Zf with regulatory elements present in the TSHβ promoter. Surprisingly, we observed that ligand (T3) weakens TR binding to a negative regulatory element (NRE) present in the TSHβ promoter. We also show that TR may interact with GATA2-Zf in the absence of ligand, but T3 is crucial for increasing the affinity of this complex for different GATA response elements (GATA-REs). Importantly, these results indicate that TR complex formation enhances DNA binding of the TR-GATA2 in a ligand-dependent manner.

**Conclusions:**

Our findings extend previous results obtained *in vivo*, further improving our understanding of how liganded nuclear receptors down-regulate gene transcription, with the cooperative binding of transcription factors to DNA forming the core of this process.

## Introduction

The molecular mechanisms that control gene expression are still emerging, revealing regulatory circuits that permit a finely tuned transition between activation and repression. We are now beginning to understand how genes are switched on and off, regulating the transcriptional activity, however the subtleties of protein-DNA interaction that drive up- or down-regulation remain elusive, especially in what concerns genes under nuclear receptor control.

The human nuclear receptor (NR) gene superfamily comprises transcription factors that modulate gene expression in a ligand-dependent manner. They have an important role in growth, development and homeostasis by regulating the expression of many genes in response to small, fat-soluble molecules, such as thyroid hormones, steroids and vitamin D. The family is mainly characterized by two domains, a highly conserved DNA-binding domain (DBD) and a less well conserved ligand-binding domain (LBD).

In general, NRs can regulate gene expression by binding directly or indirectly to specific sequences in the promoter of target genes. Moreover, they can also act by interfering with transcriptional complex formation in a DNA-independent manner. The classical mechanism of NR activity proceeds via direct DNA binding at hormone response elements (HRE) in the promoter region of target genes. The simplest current model suggests that NRs in a homo or heterodimer form, typically complexed with retinoid X receptor (RXR), bind directly to an HRE. In the absence of DNA, NRs were observed in monomeric, dimeric and tetrameric forms, the latter dissociating into dimers in the presence of DNA and/or cognate ligands [1], [2].

It has however become clear that an important regulatory activity of nuclear receptors involves repression of genes in response to regulatory ligands [3]–[5]. Indeed, microarray-based gene expression profiling suggests that, for example, in the liver, the thyroid hormone T_3_ down-regulates more than twice as many genes as it activates [6]. However, the mechanism used by a ligand-bound NR leading to repression of transcription is still a subject of contention [7].

The term “negative regulation” has been used in the context of nuclear receptor signalling to describe the ability of NRs to down-regulate transcription of a target gene via binding to a potential “negative response element” or “negative regulatory element (NRE)”. Early studies found that thyroid hormone receptor down-regulates transcription of thyrotropin α (TSHα) [8] and the thyrotropin β (TSHβ) gene [9] in response to thyroid hormone. These studies identified a so-called negative thyroid hormone response element (nTRE) downstream of the TATA box of the TSHα gene and in the first exon of TSHβ gene.

**Figure 1:**
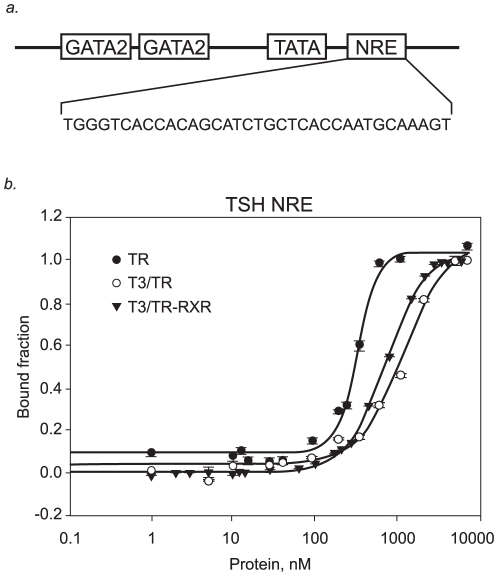
Thyroid Hormone Receptor (TR) on negative regulatory element (NRE) in TSHβ promoter. **A** – Schematic structure of the TSHβ promoter: The binding sites for GATA2, TATA box, and reported NRE. **B** – Fluorescence anisotropy curve of binding TR DL (black dots), T3/TR DL (white dots) and T3/TR DL+RXR DL to fluorophore-labelled sequence TSH NRE (black triangles). Protein titrations were made from 1 to 10000nM on 10nM of fluorescence.

In fact, the sequence in the promoter of TSHβ gene responsible for mediating inhibition by T3/TR contains a single half-site-like sequence (GGGTCA) and is denoted as nTRE [9]–[14] or NRE [15], [16] (Fig. 1a).

Thus these early models of down-regulation by nuclear receptors and other later studies [17] concluded that the liganded receptor was binding directly to DNA and interfering with the process of transcriptional activation that would normally take place in the absence of liganded nuclear receptor protein.

Some years later the requirement for DNA-binding activity in TR for the negative regulation of TSH by thyroid hormone was demonstrated *in vivo*. A “knock in” mouse harbouring a TRβ mutant defective in DNA binding was studied [18]. In these mice, thyroid hormone fails to suppress TSH gene transcription, supporting the conclusion that negative regulation of the TSH gene requires DNA binding by TR. Therefore, the direct binding to DNA seems to be one of the strategies adopted by nuclear receptors for transcriptional down-regulation. In subsequent years, however, other mechanisms used by liganded NR to down-regulate target genes have been reported, suggesting that NR does not require DNA-binding to down-regulate gene transcription.

Several models have been proposed to explain the crosstalk between NRs and general transcription factors (TF) to drive transcriptional repression. For example, applying different in vivo strategies, a model was proposed for repression by GR which involves tethering, or direct binding of the receptor to an other transcription factor [19], notably AP1 [20] and NF-κB [21]. In this trans-repression mechanism, just one transcription factor binds to the DNA, either a NR or a general TF, without the requirement of two different binding sites. Although subject to controversy, the DBD of nuclear receptors seems to be required for gene trans-repression [22]–[24].

The crosstalk between TFs, like nuclear receptors and GATA, has been reported as crucial in controlling gene expression [25]–[30]. Each of the six identified GATA proteins contains a highly conserved DNA-binding domain consisting of two zinc fingers. These two zinc fingers have been shown to bind directly to the DNA sequence element (A or T) GATA (A or G) [31], although the carboxy-terminal zinc finger is sufficient for site-specific binding [32].

More recently, an intriguing hypothesis explaining the mechanism of the negative regulation of the TSHβ gene suggests a cross talk between TR with GATA2 [16]. Using a highly sensitive reporter assay in a nonpituitary CV1 (kidney-derived) cell co-transfected with GATA2, Pit1 (another transcription factor) and/or TR, Matsushita *et al.* showed that GATA2 alone could activate the TSHβ promoter, and this transactivation was repressed by liganded TR [16]. They also demonstrated *in vivo*, by co-expression in 293T cells, and *in vitro* (GST pull-down assay) that the Zn-finger region of GATA2 (GATA2-Zf) interacts with the TR DBD in a ligand-independent manner, and this complex was essential for negative regulation by thyroid hormone. It was proposed that TR interacts with GATA2-Zf on a GATA response element (GATA-RE) present in the TSHβ promoter [16]. Furthermore, this study demonstrated in CV1 cells that the negative regulation by liganded TR was preserved after complete destruction of the NRE. Thus, contrary to previous study [15] it was suggested that the NRE, which contains a single half-site like sequence (GGGTCA), is not required for the TSHβ down-regulation.

The study of the TR-GATA2 complex on DNA is particularly attractive because it is potentially the primary complex assembled on the TSHβ promoter triggered by T3 binding to TR. In addition, the interaction of GATA2 with TR occurs through the highly conserved zinc-finger DNA binding domains. Taken together, the understanding of how T3 signalling is propagated through the TR DBDs to GATA2-Zn and DNA would provide new insights into the mechanism of gene down-regulation by a liganded nuclear receptor.

Therefore, in an effort to better understand the mechanism responsible for TSHβ down-regulation mediated by liganded TR-GATA2 complex, we have carried out equilibrium binding assays to investigate the ability of TRβ1 and GATA2-Zf to bind to different response elements in TSHβ promoter sequence.

## Results

### Thyroid Hormone Receptor (TR) on negative regulatory element in TSHβ promoter (NRE)

To enable a better understanding of the dynamic of the TR-NRE complex, we measured the binding affinity of TRβ1 DL for the fluorophore-labelled reporter sequence using direct fluorescence anisotropy titrations. Fluorescence anisotropy, based on the property of fluorescent molecules to retain the polarization of the excitation light, reflects the tumbling rate of molecules in solution. It is ideal for studying protein–DNA interactions, as the complex formed is larger and tumbles more slowly than the unbound oligonucleotide.

Therefore, we designed the anisotropy binding titration assays using the NRE present in the TSHβ promoter (TSH NRE) [15] to detect the TRβ1 binding to this negative response element in terms of dissociation constants (Kds). A Hill coefficient “n” higher than unity is indicative of a high degree of cooperativity, meaning that the binding of the first monomer facilitates the binding of the second monomer.

It has been reported that TR may bind to DNA as a monomer, homodimer or heterodimer form with RXR [33]–[35], depending on the architecture of the TRE. To verify the role of RXRα in TRβ1-TSH NRE complex, we also titrated in TR-RXR complex.

We found that in the absence of T3, TRβ1 cooperatively binds to TSH NRE with a relatively high affinity, Kd of 316+/−29 nM (n = 3.5). The Hill coefficient (n) of 3 rules out a simple monomer interaction and implies a dimerization energetically coupled to DNA binding in the nanomolar range. Surprisingly, we observed that liganded TRβ1 bound to TSH NRE (Kd = 1180+/−226 nM; n = 1.7) much weaker than the unliganded TRβ1. Importantly, liganded TRβ1 also binds to TSH NRE in a cooperative manner (Fig. 1b) (Table 1).

**Table 1:** Dissociation constants of TR and RXR binding to DNA.

	DNA			
	TSH NRE		DR4	
Protein	Kd (nM)	Hill coef. (n)	Kd (nM)	Hill coef. (n)
TR DBD-LBD	316±29	3.5±0.1	2044±266	1±0.1
T3/TR DBD-LBD	1180±226	1.7±0.4	1975±295	2±0.4
T3/TR DBD-LBD+RXR	720±19	1.8±0.1	39.4±2.2	3.7±1

Unliganded or liganded TRβ1 DL (TR DBD-LBD) and liganded TRβ1 DL+RXRα complex to fluorophore-labelled DNA (DR4). N is the Hill coefficient.

We observed that the T3/TRβ1-RXRα complex bound to NRE with a Kd of 720+/−19 nM (n = 1.8), however T3/TRβ1-RXRα binding was still 2 fold weaker than the unliganded TRβ1 binding.

### Thyroid Hormone Receptor on positive regulatory element (DR4)

In order to compare our findings on NRE with a canonical positive response element (pTRE), we designed new isothermal binding assays using DR4 (direct repeats spaced by 4 nucleotides) to detect the TRβ1 DL preferences for this pTRE in terms of dissociation constants (Kds).

**Figure 2:**
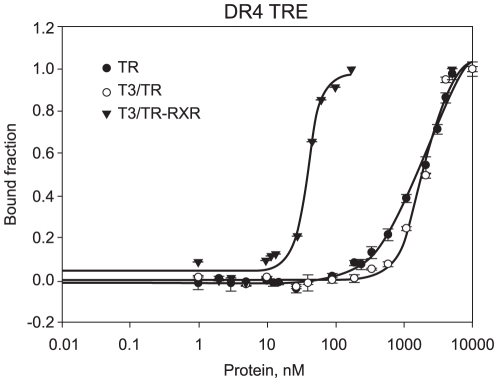
Thyroid Hormone Receptor on positive regulatory element (DR4). Fluorescence anisotropy curve of binding TR DL (black dots), T3/TR DL (white dots) and T3/TR DL+RXR DL to fluorophore-labelled sequence DR4 (black triangles). Protein titrations were made from 1 to 10000nM on 10nM of fluorescence labelled DR4.

We showed that unliganded TRβ1 DL bound weakly to DR4 (Kd = 2044+/−266 nM; n = 1.0). In presence of T3, TRβ1 revealed a somewhat similar affinity for DR4 (Kd = 1975+/−295 nM; n = 2.0), however, this binding showed a stronger tendency to form homodimers. In previous studies [36], [37], we observed that liganded TRβ1 in complex with RXRα binds more strongly to DR4 in a clearly cooperative binding (Kd = 39.4+/−2.2 nM; n = 3.7) (Fig. 2) (Table 1). Therefore, it is shown that the cooperation between RXRα DL and TRβ1 DL increases the affinity of the protein complex for the DR4 by a magnitude of 50 fold, different of RXRα DL on the NRE.

In another study, we observed that unliganded TR strongly bound (KD = 63 nM) to a longer RE containing DR4 in a cooperative way [38], suggesting TR may be better accommodated when this RE has a longer fragment flanking the DR4. Also, the 4 nucleotides between the direct repeats may have an important role for the binding affinity of TR, since they were different from the short DR4 we studied here.

### TR DBD binding to GATA2 - Zinc finger

It had previously been proposed that the negative regulation of TSHβ gene by thyroid hormone was mainly due to the interaction between TR and GATA2 [16]. It was demonstrated that TR DBD interacts with GATA2-Zf in a T3-independent manner. In this model, a TR-GATA2 is recruited to a GATA-RE to mediate the TSHβ down-regulation.

**Figure 3:**
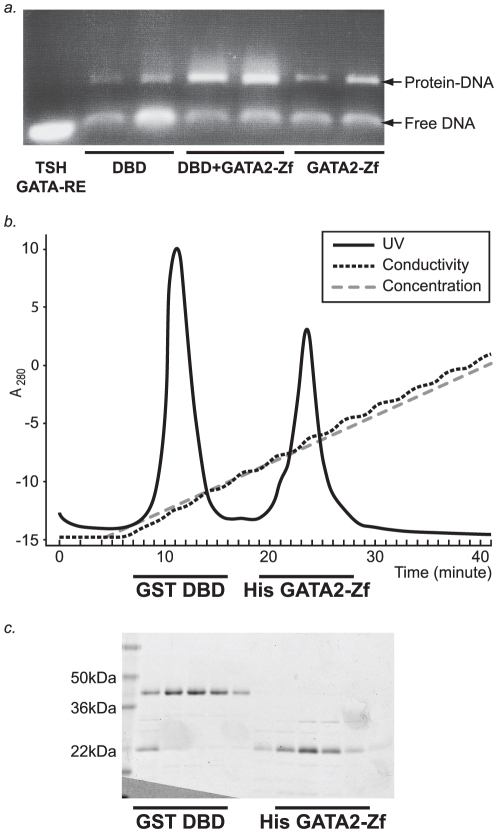
TR DBD binding to GATA2 - Zinc finger. **A** – Electrophoretic analysis of fractions purified from gel filtration: TR DBD, TR DBD+GATAZf and GATA2-Zf bound to TSH GATA-RE. The proteins alone or heterodimer TR DBD-GATA2-Zf was prepared mixing equimolar quantities (100uM) of each protein plus DNA and loaded in a gel filtration column. Fractions of the peaks eluted from gel filtration containing TR DBD-DNA, GATA2-DNA, or TR DBD- GATA2-Zf-DNA were run in agarose gel 1.3% with 0.5×TBE. Gel was stained with Ethidium bromide and analysed on the UV light. **B** and **C** – Purification of GST-TR DBD and his-GATA2-Zf in presence of TSH GATA-RE using ion exchange column. The complex TR DBD+GATA2-Zf+TSH GATA-RE was applied onto an ion exchange column, which eluted as two peaks, one corresponds to GST TR DBD and another corresponds to GATA2-Zf. The SDS-page presents each peak after purification. In lane 1 shows the molecular weight markers; lane 2 shows the two proteins (GST-TR DBD+his-GATA2-Zf) co-eluted; lanes 3 to 6 corresponds to GST-TR DBD elution; and lines 7 to 11 corresponds to GATA2-Zf elution.

To investigate the direct interaction of TR DBD with ZnFinger GATA2, we used GST-TR DBD to pull down the His-tagged GATA2-Zf (data not shown). To further characterize the potential complex, TR DBD-GATA2-Zf in the presence of a GATA-RE from the TSHβ promoter was subjected to size exclusion purification by gel filtration. However, we observed that the two proteins do not co-migrate (data not shown). The electrophoretic analysis of fractions purified from gel filtration shows that both proteins can bind to the DNA, but it was inconsistent with a heterodimer formation of GATA2-ZF with TR DBD on the DNA (Fig. 3a).

In another attempt to isolate the heterodimer complex, GST-TR DBD and His tag GATA2-Zf plus DNA were purified using an anion exchange column. Here, we found that the majority of the complex falls apart during this purification method (Fig. 3b and 3c).

**Figure 4:**
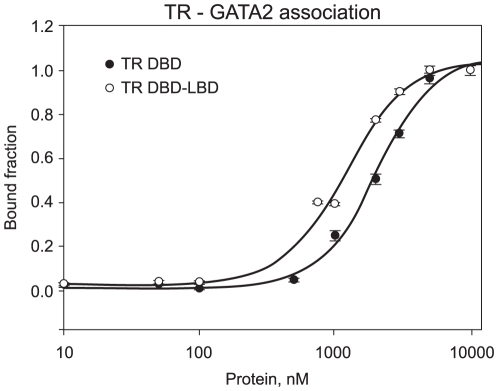
TR DBD and DL binding to GATA2 - Zinc finger. Fluorescence anisotropy curve of association of unliganded TR DL and TR DBD to fluorescein-labeled GATA2-Zf (black dots). In these curves, TR DL and TR-DBD were titrated, separately, on 10nM fluorescent probed GATA2-Zf, in a concentration range between 1 to 10.000nM.

To determine the precise binding affinity of TR for GATA2-Zf and to verify the importance of the ligand binding domain of TR in the complex formation, we performed fluorescence anisotropy assays with fluorophore-labelled GATA2-Zf and non-labelled recombinant TRs, one is TR DBD-LBD which has the DNA binding domain and ligand binding domain (TR DL) and the other is TR DBD which has just the DNA binding domain. TR DL bound to GATA2-Zf with a Kd of 1173+/−85 nM (n = 1.6), and TR DBD bound with a Kd of 1930+/−212 nM (n = 1.7) (Fig. 4). Apparently, the LBD also plays a role in this interaction, since the TR DL bound slightly stronger to GATA2-Zf.

### TR DL and GATA2-Zf on GATA-RE from TSHβ promoter

Cell-based assays had previously been shown that T3/TR might interact with GATA2-Zf on a GATA2 response element in the TSHβ promoter, leading to down-regulation [16].

**Figure 5:**
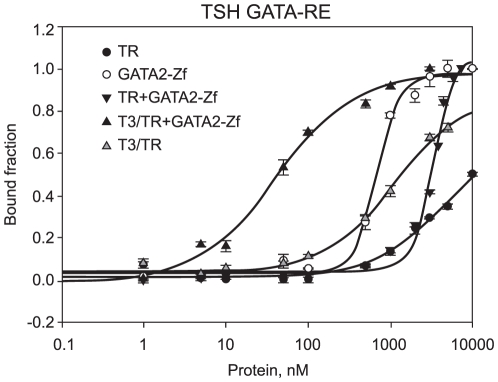
TR DL and GATA2-Zf on TSH GATA-RE from TSHβ promoter. Fluorescence anisotropy curve of binding unliganded TR DL (black dots), GATA-2 (white dots), unliganded TR DL+GATA2-Zf complex (inverted black triangles), T3/TR DL+GATA2-Zf (black triangles) and T3/TR DL (grey triangles) to 10 nM of fluorophore-labelled sequence TSH GATA-RE DNA. All curves were made separately; being each protein titrated on the DNA, in a concentration range of 1 to 10000nM.

To further investigate whether the unliganded TR and GATA2 bind to DNA in a cooperative manner, we performed new fluorescence anisotropy assays with TR DL, GATA2-Zf and TSH GATA-RE (Fig. 5) (Table 2). We observed that unliganded TR DL showed no detectable binding to TSH GATA-RE. GATA2-Zf alone bound to TSH GATA-RE with a Kd of 688+/−39 nM (n = 3.1). The association between unliganded TR DL and GATA2-Zf showed weaker binding to TSH GATA-RE, suggesting that unliganded TR inhibits GATA2 binding to DNA.

**Table 2:** Dissociation constants of TR and/or GATA2-Zf binding to DNA.

	DNA			
	TSH GATA-RE		GATA-RE	
Protein	Kd (nM)	Hill coef. (n)	Kd (nM)	Hill coef. (n)
TR DBD-LBD	ND*	ND*	ND*	ND*
T3/TR DBD-LBD	ND*	ND*	ND*	ND*
GATA2-Zf	688.8±38.7	3.1±0.4	14.9±1.5	1.3±0.4
GATA2-Zf+TR DBD-LBD	3257±160.9	3.3±0.4	1707±94.6	1.7±0.1
GATA2-Zf+T3/TR DBD-LBD	38.6±7.9	1±0.2	139.7±29.8	1±0.2

Unliganded or liganded TRβ1 DL (DBD-LBD), GATA2-Zf and unliganded or liganded TR DL+GATA2-Zf complex to fluorophore-labelled DNA (TSH GATA-RE and canonical GATA-RE). N is the Hill coefficient. ND means non determined.

**Figure 6:**
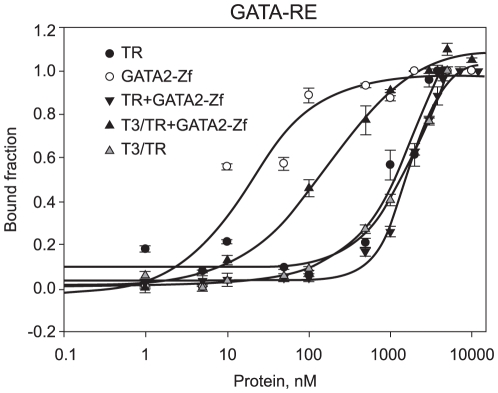
TR DL and GATA2-Zf on canonical GATA-RE. Fluorescence anisotropy curve of binding unliganded TR DL (black dots), GATA2-Zf (white dots), unliganded TR DL+GATA2-Zf complex (inverted black triangles), T3/TR DL+GATA2-Zf (black triangles) and T3/TR DL (grey triangles) to 10nM of fluorophore-labelled sequence GATA-RE. All curves were made separately; being each protein titrated on the DNA, in a concentration range of 1 to 10000nM.

To verify the specificity of GATA2-Zf binding, we used a canonical GATA-RE in the new fluorescence anisotropy assays (Fig. 6) (Table 2). As expected, GATA2-Zf bound strongly to GATA-RE. Unliganded TR DL did not show specific binding, and no Kd could be determined. Again, the association between the two proteins, unliganded TR DL and GATA2-Zf, showed weaker affinity for GATA-RE, suggesting again that unliganded TR DL may disturb the GATA2-Zf binding to GATA-RE.

In order to examine in detail the effect of T3 on TR-GATA2 binding to DNA, we performed new experiments using a canonical GATA-RE and also the TSH GATA-RE. To our surprise, T3/TR DL-GATA-Zf showed much higher affinity for both GATA-RE, TSH GATA-RE (Kd = 38.6+/−7.9 nM; n = 1) and GATA-RE (Kd = 139.7+/−30 nM; n = 1) than the complex did in the absence of T3 (Fig. 5 and 6 respectively) (Table 2). Liganded TR DL alone showed very weak binding to both GATA-REs, and we were unable to determine the precise Kd.

## Discussion

The mechanisms used by the cell to down-regulate gene transcription by liganded nuclear hormone receptors remains a conundrum in the field.

Lessons from bacteria have led to the view that higher-order complexes permit cooperative binding to both adjacent and non-adjacent operator sites. The switch between transcriptional activation and repression will depend on the formation of these higher-order protein-DNA complexes [39].

However, identification of many components involved in activation and repression of gene transcription has led us to build several models to explain how gene expression takes place in a specific cell environment. In fact, the control of the cellular concentration of transcriptional regulators appears to define and modulate gene expression [40].

Cell-based assays have suggested that GATA2 may interact with TR to down-regulate TSHβ gene transcription [16]. In trying to map the contact surface responsible for this interaction, it was proposed that the Zn-finger region of GATA2 interacts with the TR DBD in a T3-independent manner, it being sufficient to drive TSHβ down-regulation. The authors suggested that TR would bind to DNA via GATA2 on a GATA response element present in the TSHβ promoter. Challenging a previous study [15], it was suggested that a NRE is not required for this TSHβ down-regulation.

### T3 weakens the binding of TR to the NRE

As a means to address the conflicting data about the significance of NRE for TSHβ down-regulation by T3, we studied the TR binding affinity for the NRE present in the TSHβ promoter.

We found that unliganded TR binds tightly to DNA, and, we show for the first time that T3 weakens this interaction (4 fold) without affecting the cooperative nature of binding. Interestingly, RXR did increase the affinity of the liganded TR for the NRE. However, T3/TR-RXR binding was still 2 fold weaker than the unliganded TR binding. This suggests that RXR does not have a major role in the TSHβ regulation. This finding is in agreement with another study that demonstrated that RXR inhibited TR-NRE complex formation *in vitro* [15]. Also, it is in accordance with the studies on TR and RXR knockout mice, which suggest that RXR is dispensable for TSH regulation [41].

Previous studies have shown that T3 disrupts the TR dimer binding and increases the intensity of monomer binding to a TRE in a dose-dependent manner. This disruption of the TR dimer binding by T3 was believed to occur on a TRE formed by a direct repeat and not on a palindromic TRE [35]. Our data present evidence that the binding affinity of TR for DNA might be affected by modifications that occur in the LBD after ligand binding, without disrupting the oligomerization state of the protein. The structure of the full-length PPAR-RXR on DNA shows that the LBD of PPAR interacts with both the LBD and DBD of RXR and also that the hinge region makes an extensive DNA interaction. In addition, it was shown that PPAR LBD makes electrostatic interactions with DNA [42]. Taken together, it is plausible that the TR LBD plays a decisive role in the ability of the receptor to bind DNA. However, more structural information at atomic level resolution is needed to explain the precise mechanism by which T3 weakens the binding of TR to DNA.

Using mostly band shift and transient transfections assays several groups have suggested that TR interacts with an inhibitory element immediately downstream of the transcription start site, and that this interaction is important for TSHβ gene down-regulation mediated by T3 [9]–[15], [17], [43].

Employing a DNA affinity-binding assay in which endogenous proteins that bind to the NRE can be detected on a solid bead matrix, it was suggested that TRβ1 is recruited to NRE beads in a ligand-dependent manner [15]. It was also suggested that histone deacetylase (HDAC) 1 and 2 is recruited to the NRE-beads upon T3 addition, but HDAC1 was observed on NRE even in the absence of ligand. Contrarily, in the same study [15], using gel shift assay the authors observed that recombinant rTRβ1 could bind to NRE in a ligand-independent manner. The authors attributed this discrepancy to another factor postulated to be present in the extracts that can confer ligand-dependent binding of TR to the NRE. In accordance with the gel shift assay in this previous study, our findings show that unliganded TR binds to NRE. However, contrary to the results that suggested that endogenous liganded TR binds tightly to NRE, we observed that T3 attenuates TR-NRE interaction.

Conflicting with the hypothesis that T3 induces recruitment of TR to the NRE, Matsushita *et al.* [16] demonstrated that the negative regulation by liganded TR was preserved after complete destruction of the NRE, suggesting that this region is not required for the TSHβ down-regulation. In line with this, our results showing that T3 weakens TR binding to NRE may suggest that liganded TR may be displaced to another response element for which TR has higher affinity. Nevertheless, if unliganded TR binds to NRE, it is reasonable to consider that unligaded TR on NRE would be a major factor for TSHβ transcriptional up-regulation. However, there is strong in vivo evidence that conflict with the hypothesis that unligaded TR is involved in TSHβ gene up-regulation: i) TR-knockout mice over-produce thyroid hormones and thyroid stimulating hormone, indicating that activator(s) other than unliganded TR may be important for TSHβ transcriptional activation [41], [44]; ii) later analysis reported that unliganded TR is not a transcriptional activator for the TSHβ gene and that the major transcriptional activators for the TSHβ gene are Pit1 and GATA2 [16], [45]–[47]. Collectively, these indicate that unliganded TR is not involved in the TSHβ gene activation. In spite of that, we cannot exclude the hypothesis that NRE could function as a reservoir for unliganded TR, which would be readily available to act as a repressor when T3 is present. In the Matsushita et al. study [16], the need of NRE as a reservoir for unliganded TR could have been masked by the over expression of TR in the transient co-transfected cells. Therefore, it would be important to verify this last hypothesis using cell-based assays, such as chromatin IP, working with endogenous levels of receptors, or using mice models with different mutations in the TSHβ promoter.

Studies on the prokaryotic mutant Lac repressor show that a mutant repressor down-regulates *lac* genes only in the presence of lactose (inducer). The inducer weakens the protein-DNA binding, rendering the mutant capable of freeing itself from non-specific DNA binding to the *lac* operator [48]. Similar to this prokaryotic example, T3 may induce the displacement of TR from the NRE to other regulatory elements with higher affinity.

### DNA binding of the TR-GATA2 in a hormone-dependent manner

Herein, we have focused our studies on the previously described TR-GATA2 complex [16], which is the first assembled complex shown to be responsible for the down-regulation of the TSHβ gene when it is in the presence of T3. It was proposed that the interaction of the Zn-finger region of GATA2 with the TR DBD would be sufficient to drive TSHβ down-regulation.

Using different purification approaches, gel filtration and anion exchange column, we could not isolate the potential heterodimer complex, TR DBD and GATA2-Zf plus DNA. We observed that both domains could bind independently to a GATA-RE in the TSHβ promoter but could not form a stable heterodimeric complex on the DNA. Our data confirmed previous results [16] that TR DL and DBD interact with GATA2-Zf, but we showed that the affinity of TR for GATA2 is in the low micromolar range. Interestingly, TR DL bound slightly stronger to GATA2-Zf than TR DBD. This suggests that the LBD may play a role in this interaction. This phenomenon was also supported by the observation that T3/TR has a pronounced effect on GATA2 affinity for GATA-REs.

We showed that the affinity of TR-GATA2-Zf complex for GATA-RE is dependent on T3, i.e., T3 dramatically increased the affinity of TR-GATA2-Zf for GATA-RE in the TSHβ promoter. Importantly, to our knowledge these results show for the first time the hormone-dependent effect on affinity of a heterologous DNA binding transcription factor.

The fact that the T3/TR-GATA2 complex assembled leads to TSHβ down-regulation and that T3 increases the affinity of TR-GATA2-Zf for GATA-RE in the TSHβ promoter suggest that the TSHβ transcriptional down-regulation may be correlated with the TR ligand-dependent enhancement of TR-GATA2 affinity for DNA.

Indeed, studies carried out on S. cerevisiae phosphate response (PHO) gene drew attention to the correlation between DNA-binding affinity and transcriptional response. In attempt to test the hypothesis that chromatin may influence gene expression by differentially regulating the accessibility of Pho4 transcription factor sites in the PHO promoter, the authors introduced a series of variants of this promoter into haploid S.cerevisiae. This promoter contained different affinity Pho4 sites controlling transcription of a green fluorescent protein (GFP) reporter gene [49]. The authors suggested that the interplay of chromatin and binding-site affinity provides a mechanism for fine-tuning responses to the same cellular state.

In future work it would be of great significance to verify whether the increase of DNA-binding affinity of GATA2 by liganded TR directly correlates with TSHβ gene down-regulation in a chromatin context.

### Theoretical Model

**Figure 7:**
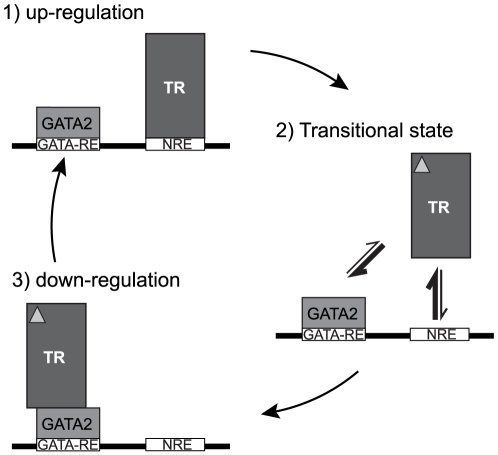
Theoretical model for TR and GATA2 state in TSHβ gene down regulation by thyroid hormone. Beside the fact that TR is not believed to participate in the up-regulation of TSHβ, the NRE may be used as a reservoir for TR, leaving the main activators, GATA2 (and, as described, Pit1), to drive gene transcription. Upon T3 binding, TR is released from NRE, favouring its translocation and interaction with GATA2-Zf on GATA-RE. The T3/TR-GATA2 complex formation is the principal complex responsible for the TSHβ gene down regulation.

Based on kinetic principles imposed by fluctuation of protein concentrations in the cell and the different affinities of these proteins to cooperatively bind to DNA, a simplified model (Fig. 7) could explain the TR and GATA2 state in the TSHβ gene down-regulation by thyroid hormone. In our model, we speculate that the unliganded TR may occupy the NRE, whilst GATA2 is on GATA-RE during the basal up-regulation. The presence of thyroid hormone, T3, weakens TR-DNA binding facilitating TR interactions with GATA2. In turn, the complex T3/TR-GATA2 has high affinity to GATA-RE, favouring an on-DNA mechanism. Therefore, the displacement of the liganded TR from NRE to another response element would be critical for the mechanism used by the cell to down-regulate TSHβ gene transcription.

In summary, we showed the equilibrium binding of TR to different regulatory elements present in the TSHβ promoter, providing a catalogue of binding affinities of TR and GATA-Zf to several enhancer elements. We observed that the ligand T3 weakens the binding of TR to NRE present in TSHβ promoter. Moreover, our data shows that T3/TR-GATA2 complex binds tightly to different GATA-RE, corroborating previous *in vivo* data that suggests this complex plays an important role for gene down-regulation in an on-DNA mechanism. Importantly, these results indicate that TR complex formation enhances DNA binding of the TR-GATA2 in a ligand-dependent manner. As the interaction of GATA2 and TR occurs through the conserved DNA binding domain, this mechanism may also be extended to other members of the nuclear receptor and GATA families.

Our data extend previous results obtained *in vivo*, further improving our understanding of how liganded nuclear receptors down-regulate gene transcription, with the cooperative binding between transcription factors and DNA forming the core of this process.

## Materials and Methods

### Protein Expression and Purification

The human TRβ1ΔAB (TR DBD-LBD or DL) construct which encodes amino acid residues 102 to 461 and the human TRβ1ΔAB/EF (TR DBD) construct that encompasses amino acid residues 102 to 198 fused in frame to the C-terminus of a poly-histidine (his) tag were expressed in the *Escherichia coli* strain BL21(DE3) using a pET28a(+) plasmid (Novagen). The modified expression and purification protocols were applied as described [2]. To produce the holo form of the receptor (TR+T3), we added T3 in 3 times molar excess (3 parts of T3 : 1 part of TR, molar concentration) and incubated the sample for 1 to 3 hour at 4°C. After incubation the TR+T3 sample was concentrated to 400uM and used as a stock protein solution for all the experiments.

The RXRα1ΔAB (DBD-LBD or DL) construct which encodes amino acid residues 126 to 462 fused in frame to the C-terminus of a poly-histidine (his) tag was expressed in the *Escherichia coli* strain BL21(DE3) using a pET28a(+) plasmid (Novagen). The modified expression and purification protocols were applied as described [50]. The heterodimer TR-RXR was prepared mixing equimolar quantities of each protein (1RXR: 1TR+T3, molar concentration) followed by purification of the complex on a gel filtration Superdex75 10/30 column (GE Healthcare). The eluted protein was checked by non-denaturing gel electrophoresis, in order to verify that only the dimer was present.

Full-length nuclear receptors are notoriously difficult to express and purify. Currently we are unable to express and purify the full-length TR construct as a soluble protein in significant amounts, and therefore we conducted our studies based on TR DBD and TR DBD-LBD (DL) constructs. It is important to mention that bacterially expressed TR DL fully reproduces the DNA binding and oligomerization properties of full length TR [2], [51].

The zinc finger region of GATA2 (GATA2 – Zf) encoding residues 291–412 was cloned from Image Clone (AU96-d11) GeneService into a pET31a (Novagen). It was expressed in the *E.coli* strain BL21 (DE3) codon plus using pet 28a (+) plasmid. A Luria Broth (LB) starter culture was inoculated with a single colony of a LB-agar culture and grown overnight at 37°C. The initial culture was inoculated at 1–5% into a larger 2XYT culture (1,6% bacto-tryptone, 1% yeast extract, 0.5% NaCl -w/v) grown at 37°C for 10h, induced with 0.5mM IPTG plus 10mM ZnSO_4_ and grow for further 6h. The induced cultures were harvested by centrifugation and pellets were ressuspended in 50mM Tris-HCl, pH8, 500mM NaCl, 1%Triton x-100, 2mM β-mercaptoethanol, 10% glycerol, 10uM ZnCl_2_ and 3mM DTT. The lysate was sonicated and centrifugated at 18000rpm for 1h, in a Sorvall SS34 rotor at 4°C. The supernatant was loaded onto Talon Superflow Metal Affinity Resin (Clontech) and was incubated for 1h at 4°C. The resin was washed in 50mM Tris-HCL, pH8.0, 300mM NaCl, 20mM imidazol, 2mM β-mercaptoethanol, 10% glycerol and 10uM ZnCl_2_ for 20cv; and the bound protein was eluted with 50mM Tris-HCL, pH8.0, 150mM NaCl, 250mM imidazol, 2mM β-mercaptoethanol and 10% glycerol. The eluted fraction was incubated with thrombin protease for 18h at 18°C. The protein was then applied to a superdex75 10/30 gel filtration column (GE Healthcare) pre-equilibrated with 20mM Hepes, pH8.0, 200mM NaCl, 10% glycerol and 3mM DTT, in order to buffer exchange. The TR-GATA2-Zf complex was prepared mixing equimolar quantities of each protein and then the complex was applied to a gel filtration superdex75 10/30 column. The eluted fractions were checked by non-denaturing gel electrophoresis, in order to verify complex formation.

The labeled GATA2-Zf containing an activated fluorescein molecule was prepared by incubating the protein and the probe, fluorescein-isothiocianate, in three times molar excess, for 4 hours at 10°C. The protein-probe complex was applied to a HiTrap desalting column (GE Healthcare), to separate the excess fluorescein from the GATA-2-fluorescein complex.

### Synthetic Oligonucleotides

The single-stranded synthetic oligonucleotides required for DNA binding studies synthesized by Sigma or Eurogenetec S.A. with an attached fluorescein molecule at the 5′ of the sense single-strand were high pressure liquid chromatography-purified. Oligonucleotides used for fluorescence anisotropy were labelled on the 5′ end of the forward strand with fluorescein-isothiociante (FITC). Only the forward DNA strand was labelled in order to avoid energy transfer between fluorophores.

The single stranded oligonucleotides, **TSHβ NRE** - 5′- TGGGTCACCACAGCATCTGCTCACCAATGCAAAGT-3′, **DR4 TRE** - 5′- AGTTCAGGTCACAGGAGGTCAGAG- 3′, **TSHβ GATA-RE** -5′- TCAATAGATGCTTTTCAGATAAGAA-3′, **GATA-RE**- 5′- CACTTGATAACAGAAAGTGATAACTCT - 3′, were paired with the complementary strand. Oligonucleotides were annealed in a PCR apparatus (Perkin-Elmer 480 programmable thermocycler, Perkin-Elmer, USA) at equimolar concentrations in water, by heating at 100°C for 10 min and slowly cooled to 25°C. Annealed oligonucleotides were stored in water at −20°C. DNA concentrations were measured by A260 (1A260Z50 mg/ml dsDNA) and at A490 nm (fluorescein, 3490Z65,000 MK1 cmK1).

### Electrophoretic analysis of TR DBD and GATA2-Zf on GATA-RE fractions purified from gel filtration

In order to analyse a potential complex formation of TR DBD and GATA2-Zf on the TSH GATA-RE, the proteins alone or the heterodimer TR DBD-GATA2-Zf were prepared by mixing equimolar quantities (100uM) of each protein plus DNA. After, an additional size exclusion purification was performed using a Superdex 75 26/60 column preparative gel filtration column in 10 mM Tris-HCl pH 8, 100 mM NaCl, 5% Glycerol and 3mM DTT, or 1×PBS, 1% Triton 100, 3mM DTT, 10uM ZnCl2.

Fractions from gel filtration containing TR DBD-DNA, GATA2-DNA, or TR DBD- GATA2-Zf-DNA were run in agarose gel 1.3% with 0.5×TBE. The gel was stained with Ethidium bromide and analysed using UV light.

### Purification of GST-TR DBD, His GATA2-Zf, and GATA-RE using ion exchange column

The human TRβ1ΔAB/EF (TR DBD) construct that encompasses amino acid residues 102 to 198 was cloned into a pGEX vector (GE Healthcare). The sequence was fused in frame to the C-terminus of a GST tag and expressed as described above. In order to isolate the described complex, equimolar quantities (100uM) of both GST-TR DBD and His-GATA2-Zf in the presence or absence of DNA were purified using a HiTrap TM SP FF ion exchange column (Pharmacia Biotech). A gradient was performed using phosphate buffer pH 6.8, 2mM DTT, 1% glycerol, and 0.1M – 2M NaCl, at a flow rate of 5mL/min. The eluted fractions were analysed by SDS-PAGE.

#### Fluorescence Anisotropy

Isothermal DNA binding assays with the TR and GATA-2 were carried out using an ISS-PC1 spectrofluorimeter (ISS, Champaign, IL), assembled in “L” geometry. Excitation was set to 480 nm, and emission at 520 nm was recorded through an orange short wave cut-off filter OG515 (cut-off 50% at 515 nm). Anisotropy values were calculated as described in previous studies [38]. All fluorescence anisotropy data shown in this study are an average of at least three independent experiments, performed with different protein batches and at different times.

#### Titrimetric Assay of Protein – DNA Interaction

Isothermal assays of DNA binding to TR DBD-LBD, RXR DBD-LBD, TR+RXR DBD-LBD, GATA-2, TR+GATA-2 were performed by titrating a fixed amount of DNA-FITC (10nM) with varying amount of protein, in a concentration range of 1nM to 10.000nM (concentrations expressed as monomers in case of TR, RXR and GATA and in oligomeric form in case of heterodimer TR+RXR or GATA-2+TR complex). In all cases, maximal dilution was less than 20%. Changes in fluorescence intensity and in the DNA or protein concentrations were corrected for dilution. Binding reactions were carried out at 10°C in 50mM NaCl, 20mM Hepes pH8.0, 3mM dithiothreitol, 5% glycerol. These assays were made in presence of 1mM MgCl_2_ to avoid non-specific binding. The protein-DNA interaction isotherms were analyzed considering, firstly, a simple two-state reversible equilibrium between protein and DNA, and, secondly, using the Hill approach. The dissociation constant K_d_ of the above reaction can be described according to:
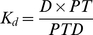
(3),where D is the molar concentration of the free double-stranded DNA binding site, PT is the molar concentration of the free protein, and PTD is the molar concentration of the complex formed between protein and DNA. The complex is related to the anisotropy measurements by:
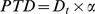
(4),and
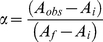
(5),where α is the fraction of bound DNA, A_obs_ is the observed anisotropy at total monomeric protein concentration PT, and A_i_ and A_f_ are, respectively, the lower and upper asymptotic limits for anisotropy values obtained by fitting the curves. Then, it follows that
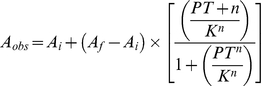
(6),where *n* is the Hill cooperativity coefficient [52]. No corrections were applied to quantum yields since no significant changes in fluorescence intensity occur through the binding curves. All equations were adjusted to the data by nonlinear least-squares regression using SigmaPlot 2006 (version 10.0, Jandel Scientific Co.).

#### Titrimetric Assay of Protein – Protein Interaction

These assays were performed in order to analyze a potential interaction between TR and GATA-2 [16]. Isotherm binding assays were performed by incubating 10nM of fluorescein-labeled GATA2-Zf with unlabeled TR DBD-LBD or TR DBD in the concentration ranging from 1nM to 10.000nM. The assays were performed at 10°C in 50mM NaCl, 20mM Hepes pH8.0, 3mM dithiothreitol, 5% glycerol. TR-GATA-2 binding assays were analyzed considering the simple two-state reversible equilibrium between TR as described elsewhere [53].

High Nuclear Receptor concentration in low salt conditions favours nonspecific binding to DNA [54], imposing a limitation of finding a second plateau in fluorescence anisotropy assays. Here the concentrations of NRs were kept below 10uM to avoid nonspecific binding titration analyse. In order to better define the second plateau of DNA saturation, measurements indicating nonspecific binding were ignored and the last binding point was repeated 3 times at its original concentration, thus fixing the second plateau and allowing Kds to be recalculated. The Kd values did not change significantly and the errors referring to Kd determinations were lower than the first approach. In this way, we confirmed the first Kd values and achieved our second plateau.
